# Elucidation of crystal and electronic structures within highly strained BiFeO_3_ by transmission electron microscopy and first-principles simulation

**DOI:** 10.1038/srep46498

**Published:** 2017-04-19

**Authors:** In-Tae Bae, András Kovács, Hong Jian Zhao, Jorge Íñiguez, Shintaro Yasui, Tomohiro Ichinose, Hiroshi Naganuma

**Affiliations:** 1 Small Scale Systems Integration and Packaging Center, State University of New York at Binghamton, Binghamton, New York 13902, USA; 2 Ernst Ruska-Centre for Microscopy and Spectroscopy with Electrons (ER-C), Peter Grünberg Institute, Forschungszentrum Jülich, Jülich 52425, Germany; 3 Materials Research and Technology Department, Luxembourg Institute of Science and Technology (LIST), 41 rue du Brill, L-4422 Belvaux, Luxembourg; 4 Laboratory for Materials and Structures, Tokyo Institute of Technology, 4259-J2-43, Nagatsuda-cho, Midori-ku, Yokohama, 226-8502, Japan; 5 Department of Applied Physics, Graduate School of Engineering, Tohoku University, Sendai 980-8579, Japan; 6 Unit´e Mixte de Physique, CNRS, Thales, Univ. Paris-Sud, Universit´e Paris-Saclay, 91767 Palaiseau, France

## Abstract

Crystal and electronic structures of ~380 nm BiFeO_3_ film grown on LaAlO_3_ substrate are comprehensively studied using advanced transmission electron microscopy (TEM) technique combined with first-principles theory. Cross-sectional TEM images reveal the BiFeO_3_ film consists of two zones with different crystal structures. While zone II turns out to have rhombohedral BiFeO_3_, the crystal structure of zone I matches none of BiFeO_3_ phases reported experimentally or predicted theoretically. Detailed electron diffraction analysis combined with first-principles calculation allows us to determine that zone I displays an orthorhombic-like monoclinic structure with space group of *Cm* (=8). The growth mechanism and electronic structure in zone I are further discussed in comparison with those of zone II. This study is the first to provide an experimentally validated complete crystallographic detail of a highly strained BiFeO_3_ that includes the lattice parameter as well as the basis atom locations in the unit cell.

BiFeO_3_ (BFO) has been known as a multiferroic material with ferroelectricity and essentially *G*-type antiferromagnetism^1^,^2^, which has application potential for emerging spintronics technology such as multiple-state memory and magnetic random access memory. While its magnetoelectric response was found too weak for practical device application^1^,^3^ in the past, recent studies using high quality single crystalline bulk BFO^4^,^5^ have revealed the true polarization value is one order of magnitude higher than previous thought, i.e., ~60 μC cm^−2^. Besides, with the availability of high quality single crystalline oxide substrates, BFO thin films grown epitaxially have shown significantly increased polarization values around 90–115 μC cm^−2^ 6,7,8,9. Since the crystal structure and/or lattice parameters of the substrates are different from those of BFO material, BFO thin films are expected to be under lattice stress/strain, which is closely related to increased polarization values as well as other physical properties in epitaxial BFO thin films. Thus, considerable experimental and theoretical efforts have been devoted to understand the lattice stress/strain effects on epitaxial BFO films that present lattice distortions found in rhombohedral unit cell of bulk BFO^6^,^10^,^11^,^12^ and also different BFO crystal unit cell structures^13^,^14^,^15^,^16^,^17^,^18^,^19^,^20^. Recently, it was suggested that, while BFO thin films are likely to possess tetragonal and/or monoclinic structures (denoted as M_*C*_ and M_*A*_) under compressive stress, they rather grow as orthorhombic and/or monoclinic structures of a different type (M_*B*_) under tensile stress^21^. However, the precise crystal models that can explain experimentally found highly stressed BFO are rather unclear as has been pointed out very recently^22^,^23^. Most of the experimental reports dealing with strain effects on epitaxially grown BFO films are making discussions based on lattice parameter changes and/or unit cell distortion^6^,^10^,^11^,^12^,^13^,^14^,^15^,^16^,^17^,^18^,^19^,^20^. On the other hand, it is worth noting that when those lattice parameter changes and/or unit cell distortions occur in epitaxially grown BFO films, *locations of basis atoms* in the unit cell change as well, which lead to corresponding alteration in its reciprocal space in terms of locations, symmetries, and shapes in Bragg’s reflections. In particular, for a material like BFO consisting of multiple elements, the alteration in the reciprocal space can be more dramatic in that slight changes in the location of each constituent atom can readily cause extra Bragg’s reflections.

In order to address this challenging issue adequately, a careful investigation on wide range reciprocal space information, i.e., *Q* (scattering vector) of >200 nm^−1^, is required to be compared with structure factor calculation results of possible crystal models (either simulated by first-principles theory or derived by powder x-ray diffraction technique) that provide not only lattice parameter but also *locations of all the basis atoms* in the unit cell. To the best of our knowledge, no previous experimental reports have discussed about this except for those dealing with bulk BFO crystal structure^24^,^25^.

In this study, a comprehensive approach combining aberration-corrected transmission electron microscopy (TEM) and first-principles methods has been utilized to unveil a complete crystal structure that includes lattice parameter as well as locations of basis atoms in the unit cell. In addition, growth behavior of a highly strained BFO thin film grown on LaAlO_3_ (LAO) is discussed as well.

## Results and Discussion

**Figure 1:**
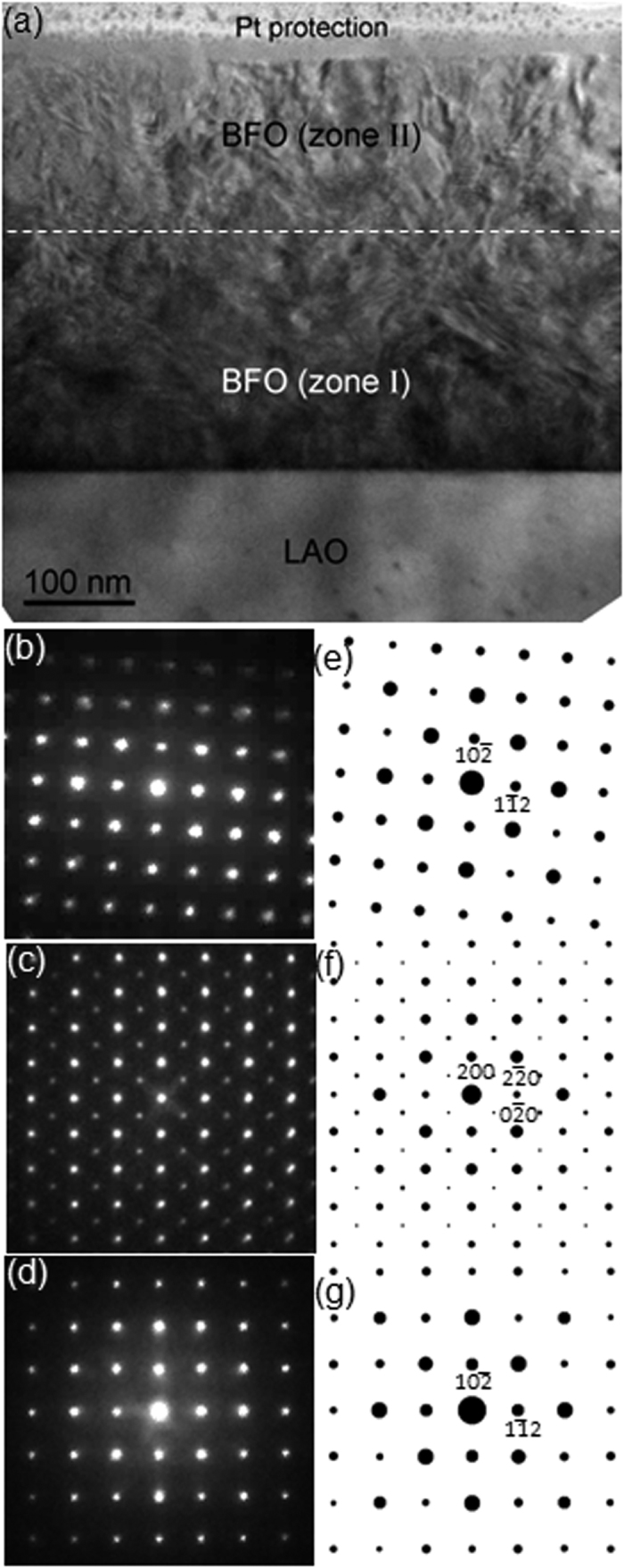
(**a**) A cross-sectional BF TEM image of ~380 nm BFO layer from [241]_LAO_ zone axis with ED patterns from (**b**) zone II and (**c**) zone I, (**d**) LAO substrate. SF calculations of the corresponding ED patterns are shown in (**e**), (**f**) and (**g**), respectively.

Figure 1(a) shows a cross-sectional bright-field (BF) TEM image of the BFO films grown on LAO substrate along the [241] zone axis. It exhibits two distinctive layers with darker and brighter contrasts denoted as zone I and zone II, respectively. In addition, both of the zones show contrasts associated with lattice defects and lattice stress. In order to acquire information about crystallographic details on the two zones, electron diffraction (ED) patterns were recorded from both of them using ~80 nm diameter of electron probe, as shown in Fig. 1(b) and (c). The boundary between zones I and II are drawn ~200 nm above zone I/LAO interface based on the ED analysis making use of ~3 nm diameter of electron probe. Note that the true boundary morphology might not be perfectly straight in atomistic scale. It will be discussed later with atomic resolution images.

**Figure 2:**
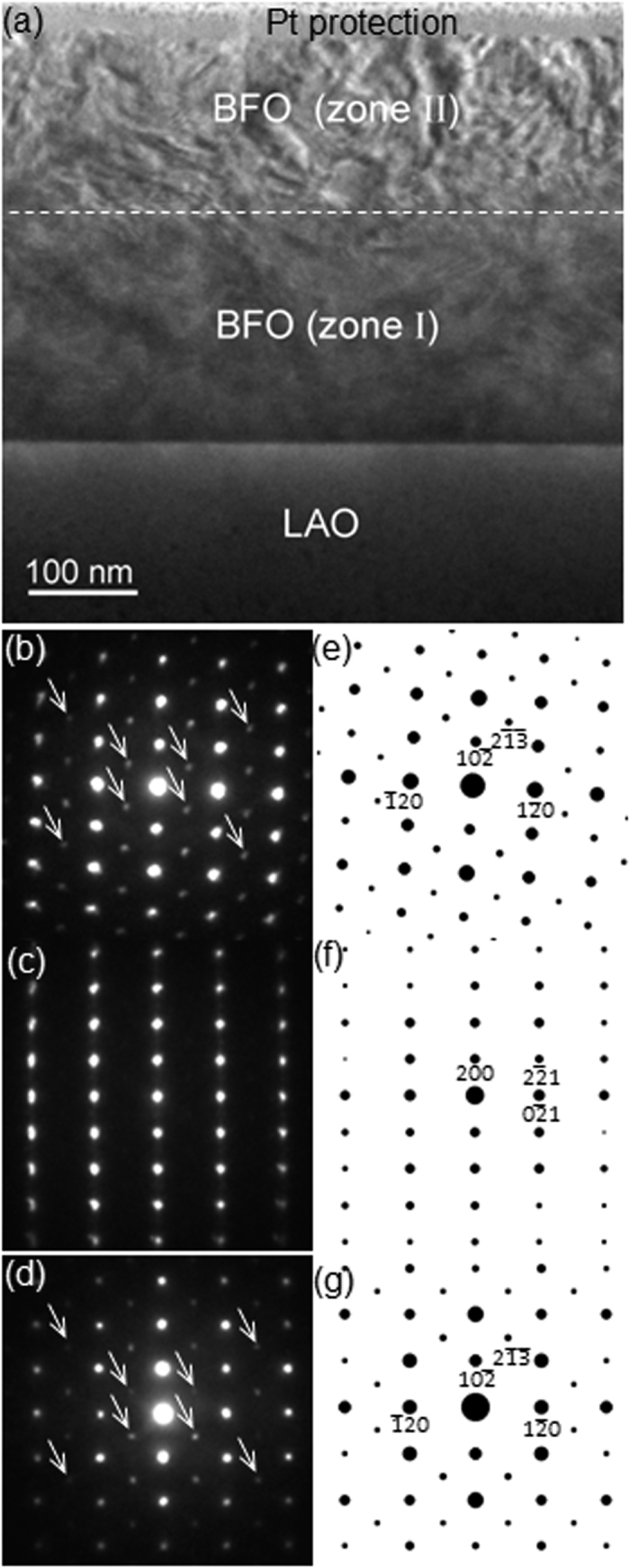
(**a**) A cross-sectional BF TEM image of ~380 nm BFO layer from [211]_LAO_ zone axis with ED patterns from (**b**) zone II and (**c**) zone I, (**d**) LAO substrate. SF calculations of the corresponding ED patterns are shown in (**e**), (**f**) and (**g**), respectively.

The ED pattern from the [241] zone axis of the LAO substrate is also obtained, as shown in Fig. 1(d), to use it as an undistorted reference material for precise camera length calibration. Note that symmetry of the Bragg’s reflections in the ED pattern from zone II (Fig. 1(b)) is identical to that in the ED pattern from LAO substrate (Fig. 1(d)), indicating that the crystal structure of BFO in zone II is rhombohedral, i.e., that of bulk BFO. Note that the orientation of Bragg’s reflection along the surface normal direction in Fig. 1(b) is ~4° off from that in Fig. 1(d). The reason will be discussed later with a high resolution TEM image. On the other hand, the ED pattern from zone I (Fig. 1(c)) is obviously different from that of zone II in terms of its symmetry as well as the locations of Bragg’s reflections, indicating a different crystal structure. As discussed in previous reports^24^,^25^, it is necessary to perform an ED analysis from another zone axis to confirm its crystal structure correctly since an ED pattern obtained from TEM is merely a two dimensional cross-section of the three dimensional reciprocal lattice of the material. Thus, the same sample was prepared for TEM observation along [211] zone axis, i. e., 45° away from [241], of LAO substrate as shown in Fig. 2. Figure 2(a) shows a BF TEM image with zones I and II whose characteristics are the same as those in Fig. 1(a). The ED patterns recorded from zone II, zone I and the LAO substrate with an electron probe of ~80 nm diameter are shown in Fig. 2(b,c) and (d), respectively. Similarly to Fig. 1, we find that, while the ED pattern from zone II (Fig. 2(b)) is the same as that from LAO substrate (Fig. 2(d)) in terms of symmetry in Bragg’s reflections, zone I is distinctively different in terms of symmetry as well as locations of Bragg’s reflections. Note that the orientations of all the ED patterns are preserved with respect to the BF images in Figs 1 and 2 to further investigate the epitaxial relationships among zone I, zone II and the LAO substrate.

For a precise ED pattern analysis, the structure factor, *F*_*hkl*_, where *hkl* represents a specific Bragg’s reflection, was calculated for all BFO phases discussed in previous reports (including theoretically predicted metastable ones) that provide all the necessary crystallographic information, including basis atom locations in the unit cell. These structures are: rhombohedral BFO (space group: *R3c, a* = 0.5678 nm, *c* = 1.3982 nm, *α* = *β* = 90°, *γ* = 120°)^18^, monoclinic BFO (space group: *P2*_*1*_*/m, a* = 0.5615 nm, *b* = 0.7973 nm, *c* = 0.5647 nm, *α* = 90°, *β* = 90°, *γ* = 90.1°)^19^, tetragonal BFO (space group: *P4mm, a* = 0.367 nm, *c* = 0.464 nm)^17^, monoclinic BFO (space group: *Pc, a* = 0.7291 nm, *b* = 0.5291 nm, *c* = 0.5315 nm, *α* = 90°, *β* = 139.46°, *γ* = 90°)^20^, monoclinic BFO (space group: *Cm, a* = 0.9354 nm, *b* = 0.7380 nm, *c* = 0.3804 nm, *α* = 90°, *β* = 86.60°, *γ* = 90°)^20^, orthorhombic BFO (space group: *Pna2*_*1*_, *a* = b = 0.5314 nm, *c* = 0.9452 nm, *α* = *β* = *γ* = 90°)^20^, monoclinic BFO (space group: *Cc, a* = 1.0604 nm, *b* = 0.5322 nm, *c* = 0.5323 nm, *α* = 90°, *β* = 62.80°, *γ* = 90°)^20^, orthorhombic BFO (space group: *Pnma, a* = 0.5650 nm, b = 0.7770 nm, *c* = 0.5421 nm, *α* = *β* = *γ* = 90°)^20^, and orthorhombic BFO (space group: *Pna2*_*1*_, *a* = 0.5702 nm, b = 0.5507 nm, *c* = 0.8036 nm, *α* = *β* = *γ* = 90°)^20^. The structure factor for LAO (space group: *R*
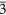
*c, a* = 0.5366 nm, *c* = 1.3110 nm, *α* = *β* = 90°, *γ* = 120°) was also calculated to investigate the epitaxial relationship between LAO and BFO overlayers^26^. The calculation of the ED patterns was based on kinematical approximation:


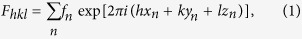


where *f*_*n*_ is the atomic scattering factor for atom *n* at fractional coordinates (*x*_*n*_, *y*_*n*_, *z*_*n*_). Details about this type of ED pattern analysis and structure factor calculation have been given elsewhere^24^. As a result, it was found that Figs 1(b) and 2(b) correspond to [241] and [211] net patterns of rhombohedral- (*r*-) BFO, confirming that zone II consists of BFO material with its *bulk crystal structure*. Note that the Bragg’s reflections marked with white arrows in Fig. 2(b) result from double diffraction^24^,^25^. While the ED patterns in Fig. 1(b) and (d) show a four-fold symmetry, they should not be confused with the [100] net pattern of cubic materials, as discussed previously^24^,^25^. It is worth noting that no evidence of significant distortion in BFO rhombohedral lattice was found from Figs 1(b) and 2(b). On the other hand, for Figs 1(c) and 2(c), i.e., for the ED patterns from zone I, while none of the aforementioned BFO phases was able to reproduce ED patterns that perfectly match both of Figs 1(c) and 2(c), the [001] net pattern from the monoclinic BFO from reference 20 (space group: *Cm, a* = 0.9354 nm, *b* = 0.7380 nm, *c* = 0.3804 nm, *α* = 90°, *β* = 86.60°, *γ* = 90°) interestingly match Fig. 1(b) as shown in supplementary Fig. S1(a). However, none of its net patterns match Fig. 2(c) including the [012] net pattern of which symmetry and Bragg’s reflection locations are similar but different (see supplementary Fig. S1(b)). It implies the true crystal structure in zone I could be a derivative of it. In fact, a previous study of pulsed laser deposition-grown BFO thin films on LAO showed that an ED pattern from a local BFO area matches [001] of the *Cm* phase^22^. However, no further ED study from another zone axis was performed to confirm the validity of the *Cm* phase.

In order to further explore the crystal structure of zone I, first-principles calculations were performed focusing on the above mentioned *Cm* phase of BFO. First, we considered the fixed in-plane lattice parameters of **a** = (2a_IP_, 0, 0) and **b** = (δ’, 2a_IP_, 0). Here, a_IP_ is the in-plane lattice constant of the LAO substrate (~3.79 Å), and δ’ is about −0.013 Å. This is to mimic the non-90-degree angle of LAO’s in-plane lattice vectors (~90.1°). Then, we fully optimized the out-of-plane lattice vectors of *Cm* BFO, which is shown as **c** = (δ_1_, δ_2_, 2a_IP_+δ_3_), as well as the atomic positions. This case is denoted as case (1) in our theoretical study. Note that, here, all δ_1_, δ_2_ and δ_3_ are optimized. Furthermore, we also simulated the *Cm* phase of BFO on the LAO substrate by fixing the in-plane lattice vectors (of the *Cm* phase) as **a** = (2a_IP_, 0, 0) and **b** = (0, 2a_IP_, 0). Note that here we maintained the angle between two in-plane lattice vectors as 90° (which is rather close to such angle of LAO substrate, ~90.1°), to mimic a square substrate. Then, we optimize the out of plane lattice vectors of *Cm* BFO **c** = (δ_1_, δ_2_, 2a_IP_+δ_3_) considering two strategies: (i) optimizing all δ_1_, δ_2_ and δ_3_, and (ii) fixing δ_1_ and δ_2_ as 0 and optimizing only δ_3_. The latter strategy corresponds to the limit of thin films in which the out-of-plane lattice vector is forced to be perpendicular to the substrate. Strategies (i) and (ii) are denoted cases (2) and (3), respectively, in addition to the above mentioned case (1). These three cases will allow us to fully compare the experimental TEM images with simulation results for the *Cm* phase. Structure factor calculations for all three cases, followed by careful comparison with the ED patterns, lead to a conclusion that only *case* (3) reproduces a *Cm* phase that immaculately explains Figs 1(c) and 2(c). The crystallographic details of the *Cm* phase are shown in Table 1. (Note that although symmetry of basis atom locations in the unit cell has monoclinic, i.e., *Cm, α, β* and *γ* angles are ~90°. Thus, hereafter the *Cm* BFO phase shown in Table 1 is termed as orthorhombic-like monoclinic.) For example, the ED patterns in Figs 1(c) and 2(c) turned out to match [001] and [012] net patterns of the *Cm* phase in Table 1 in terms of symmetry and locations of Bragg’s reflections, as revealed in their corresponding structure factor calculations shown in Figs 1(f) and 2(f), respectively. This suggests that the first-principles approach adopted in the present study successfully generates the crystal structure of BFO in zone I. Based on the results shown in Figs 1 and 2, the epitaxial relationship between zone I – i.e., *Cm* BFO, denoted as *m*-BFO hereafter – and the LAO substrate is confirmed as follows:


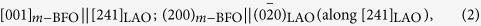



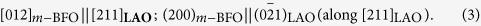


**Figure 3:**
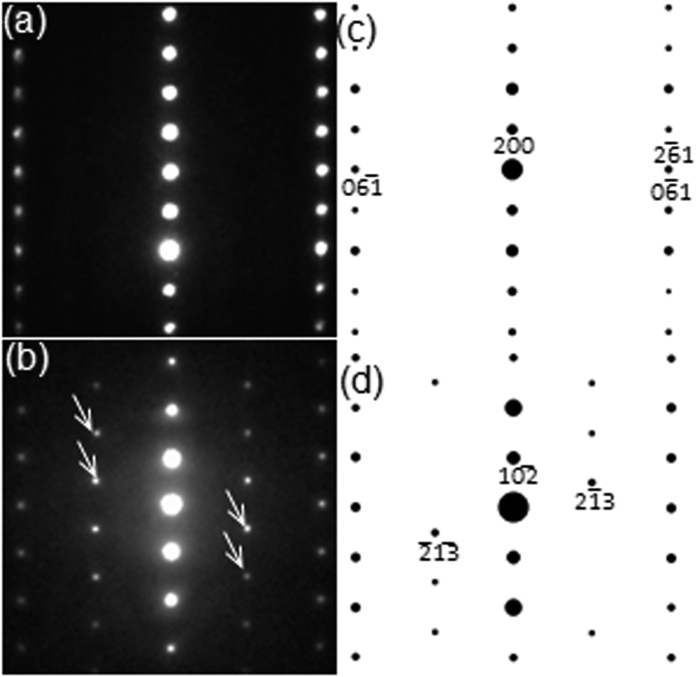
ED patterns from zone I (**a**) and LAO substrate (**b**) along [271]_LAO_ zone axis with their corresponding structure factor calculation results shown in (**c**) and (**d**), respectively.

In order to further confirm the validity of *m*-BFO model, the [241]_LAO_ cross-sectional TEM sample was tilted by ~18° within the TEM column to record the ED patterns from zone I and the LAO substrate, as shown in Fig. 3(a) and (b), respectively. Structure factor calculations shown in Fig. 3(c) and (d) clearly reveal that they correspond to [016] net pattern of the *Cm* phase and the [271] net pattern of LAO. Note that the intensity maxima resulting from double diffraction are denoted with arrows in Fig. 3(b). It is worth noting that the angle between [106] (Fig. 3(a)) and [001] (Fig. 1(c)) of the orthorhombic-like monoclinic phase is calculated to be 18.4°, which is in good agreement with the angle of 17.9° obtained between [271] (Fig. 3(b)) and [241] (Fig. 1(d)) of the underlying LAO substrate. This further confirms the validity of the orthorhombic-like monoclinic structure given in Table 1. For comparison, the [016] net pattern of the monoclinic BFO from reference 20 is calculated as shown in supplementary Fig. S1(c). At first glance, Fig. S1(c) seems similar, but it does not match Fig. 3(a) in terms of: (1) the angle between (200) and (06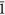) not being 90° and (2) locations of (2
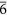
1) and (0
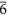
1) being different from those in Fig. 3(a).

In addition, x-ray diffraction (XRD) with *θ* − 2*θ* geometry, i.e., scanning along surface normal direction, is performed using Cu *Kα* to investigate the homogeneity of phases throughout the BFO layer as well as possible incorporation of second phases such as Bi_2_Fe_4_O_9_ and Bi_2_O_3_ (see supplemental Fig. S2). As a result, Bragg’s reflections corresponding to surface normal direction from of *m*-BFO and *r*-BFO are confirmed. Note that no Bragg’s peaks are left unexplained. This is in good agreement with the ED analyses shown in Figs 1, 2 and 3, indicating that the BFO layer is highly homogeneous consisting of *m*-BFO and *r*-BFO only with no second phase incorporation.

**Figure 4:**
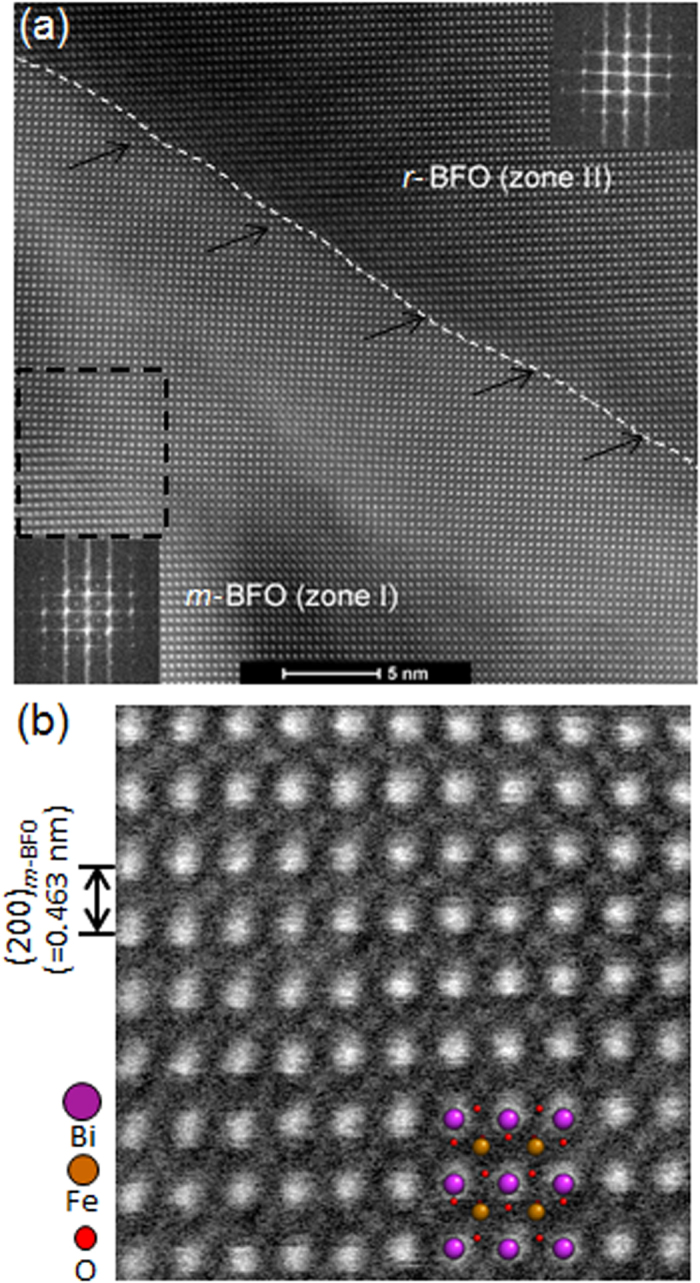
(**a**) A cross-sectional HAADF-STEM image exhibiting interface between zone I and zone II with corresponding fast Fourier transform patterns as insets. The area denoted by a square is enlarged (**b**) with superposition of atomistic model of *m*-BFO.

To provide more direct information about atomistic structural details, high-angle annular dark-field (HAADF)-scanning TEM (STEM) images were obtained from the interfaces between zone I (*m*-BFO) and zone II (*r*-BFO) along the [241]_LAO_ zone axis, as shown in Fig. 4. Figure 4(a) shows an area of lattice distortion in zone I, as denoted by a dashed square indicating that zone I is under lattice strain. Two fast Fourier transform (FFT) patterns shown as insets reveal the same characteristics, i.e., extra columns of Bragg’s reflections in *m*-BFO only, found in their respective ED patterns (Fig. 1(b) and (c)) confirming that these are not charge coupled device (CCD) camera artifacts nor reflections from higher order Laue zone, but originate from the BFO crystal structure. It is worth noting that (10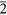) of *r*-BFO is ~3° off the surface normal direction, which is consistent with the angle of ~4° off found in Fig. 1(b). The reason for this slight tilt angle is considered to be associated with a coherent lattice planes transition at the *m*-BFO/*r*-BFO interface as denoted by arrows in Fig. 4(a). A magnified HAADF-STEM image from *m*-BFO area is shown in Fig. 4(b) with its atomistic model superimposed. While the locations of the Bi and Fe atoms match their presumed location based on the atomistic model, it is hard to locate O atoms primarily due to the much lighter atomic mass contrast of oxygen^27^. Nonetheless, it can be readily concluded that first-principles calculation, XRD, ED patterns, and structure factor calculation, combined with atomic resolution HAADF verify that BFO in zone I has the orthorhombic-like monoclinic crystal structure detailed in Table 1.

**Figure 5:**
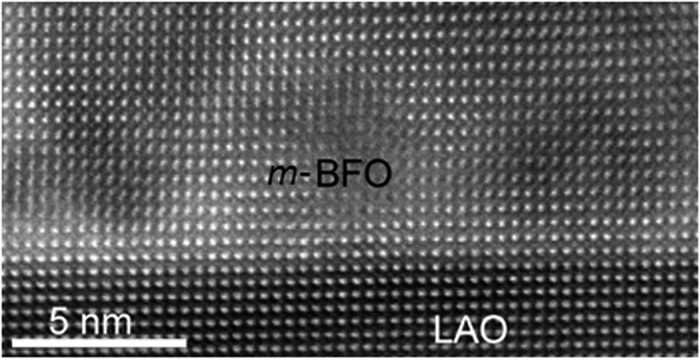
A [241]_LAO_ cross-sectional HAADF-STEM image at *m*-BFO/LAO interface showing smooth lattice plane transition from LAO to *m*-BFO with no sign of significant lattice imperfections.

**Figure 6:**
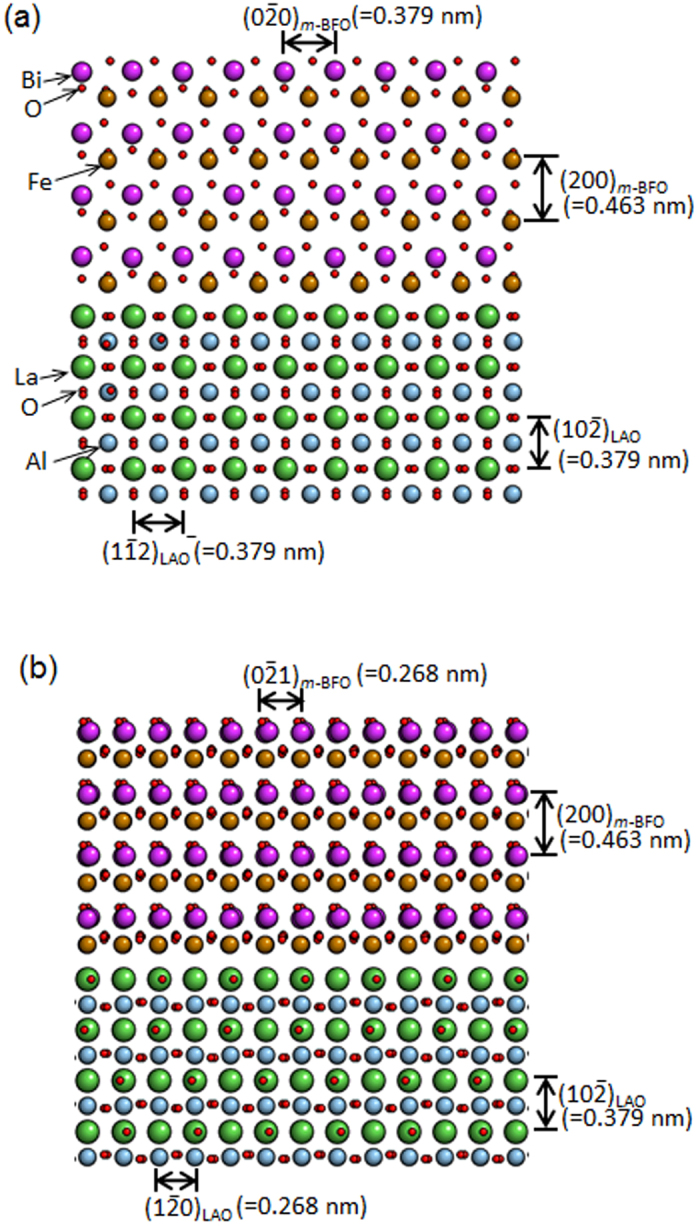
Atomistic models at *m*-BFO/LAO interface along [241]_LAO_ zone axis (**a**) and [211]_LAO_ zone axis (**b**) that shows lattice mismatch at the interface is ~0.0%.

In order to further study the *m*-BFO growth mechanism on LAO substrate, an atomic resolution HAADF-STEM image along the [241]_LAO_ zone axis was acquired from the *m*-BFO/LAO interface as shown in Fig. 5. It can be readily noticed that lattice planes run smoothly from LAO through *m*-BFO, indicating a *coherent* interface. In fact, if atomistic models between *m*-BFO and the LAO substrate are constructed based on the epitaxial relationship found above, as shown in Fig. 6, it can be noticed that the lattice spacings along in-plane direction between LAO, i.e., (1
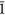
2) with 0.379 nm, and *m*-BFO, i.e., 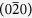 with 0.379 nm match with 0.0% of lattice mismatch along the [241]_LAO_ zone axis (see Fig. 6(a)). Similarly, when projected along the [211]_LAO_ zone axis (Fig. 6(b)) the lattice spacing match with 0.0% of lattice mismatch along the in plane direction as well. On the other hand, the lattice mismatch between *r*-BFO and LAO along the in plane direction is, for instance, 5.3% when calculated for 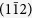 of *r*-BFO and 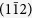 of LAO. Thus, it is believed that the minimal lattice mismatch attributed to the epitaxial relationship found between *m*-BFO and LAO is the reason that *m*-BFO, rather than *r*-BFO, starts to grow on the surface of LAO. As the *m*-BFO layer becomes thicker, however, the biaxial constraint imposed by LAO substrate should weaken. Thus, the equilibrium BFO phase, i.e., *r*-BFO, is expected to start growing on top of *m*-BFO.

**Figure 7:**
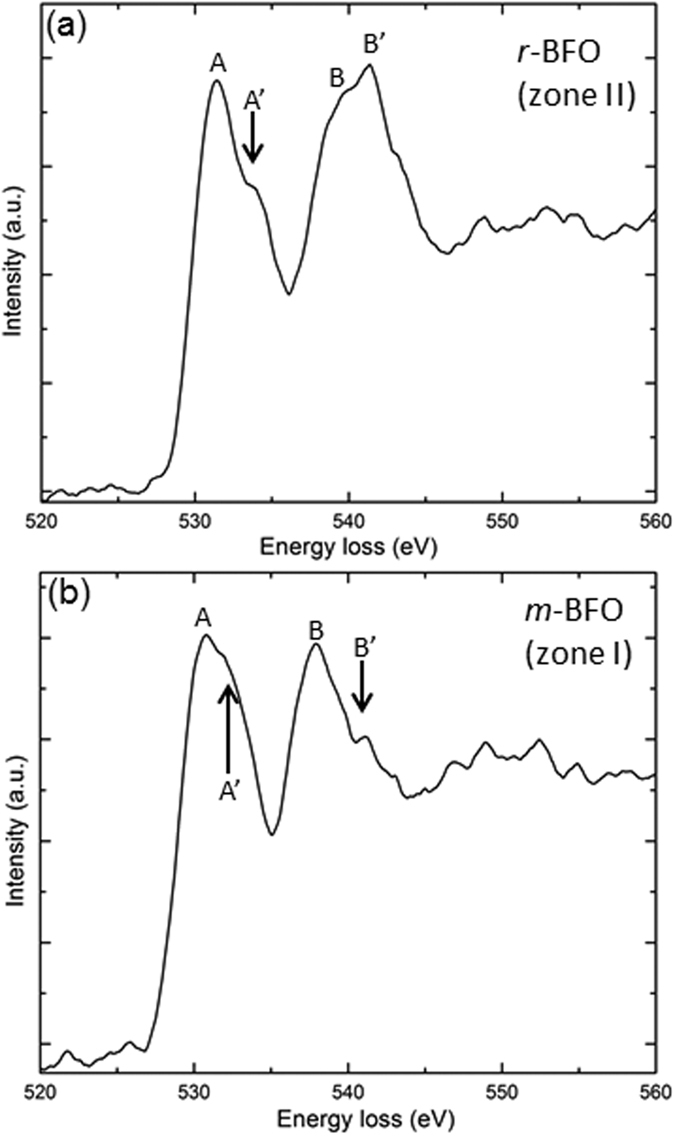
O *K*-edge EEL spectra acquired from (**a**) zone II (=*r*-BFO) and (**b**) zone I (=*m*-BFO).

**Figure 8:**
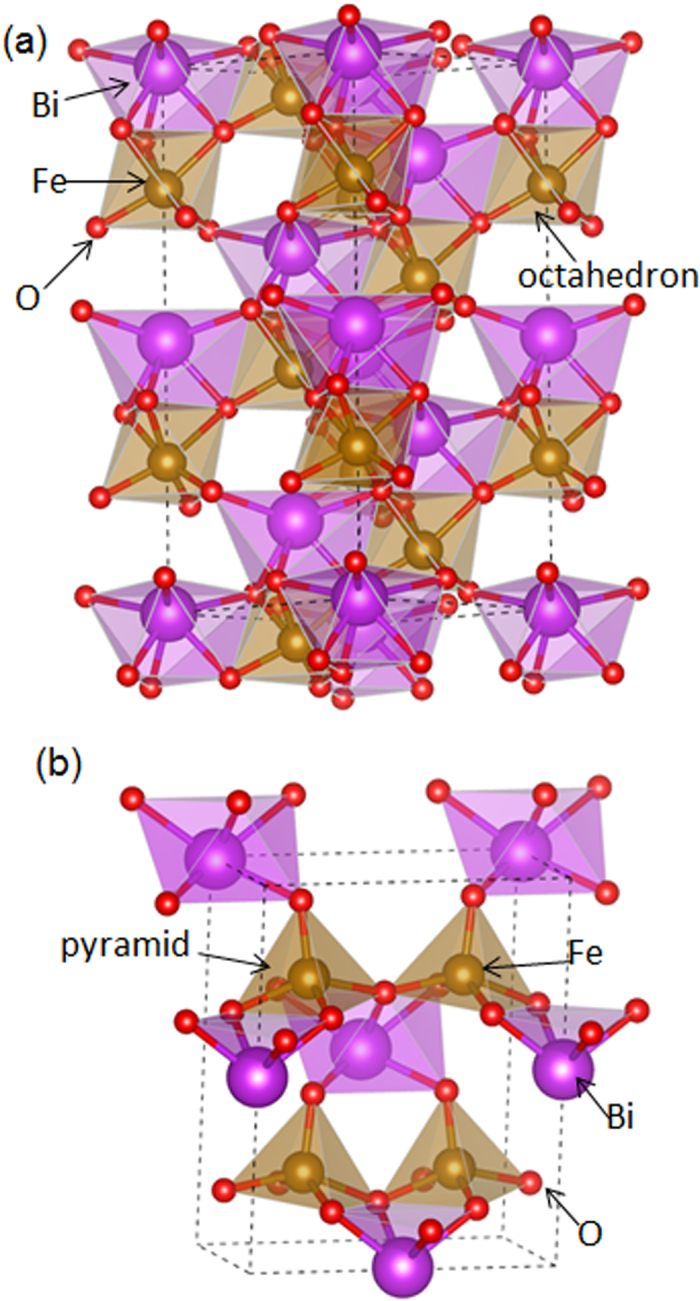
Atomistic models of (**a**) *r*-BFO and (**b**) *m*-BFO showing octahedral and pyramidal bonding geometries between Fe and O respectively.

Since BFO in zone I is confirmed a new BFO phase with space group of *Cm*, it is worth investigating its local electronic structure in comparison with that of *r*-BFO using O *K*-edge that is sensitive to local bonding and geometry. Figure 7 shows the O *K*-edge electron energy loss (EEL) spectra acquired from *r*-BFO, zone II, (a) and *m*-BFO, zone I, (b) with ~1.0 eV energy resolution. It is known that the O *K*-edge spectrum of BFO is divided in two regions, i.e., a pre-edge region ranging from ~530 to ~538 eV and a post-edge region ranging from ~538 to ~548 eV^28^,^29^. For the pre-edge region, the peaks denoted A in Fig. 7(a) and (b) are attributed to hybridization between O 2*p* and Fe 3*d* states^28^,^29^. Besides, the shoulders denoted A’ in Fig. 7(a) and (b) are considered to stem from a transition between O 2*p* and Bi 5*d* states or possibly that between O 2*p* and Bi 6*d* states in BFO^28^,^29^,^30^. On the other hand, the (sub)peaks denoted B and B’ within the post-edge region in Fig. 7(a) and (b) are known to be associated with hybridization between O 2*p* and Fe 4*sp* states. It has been found that the (sub)peaks split with 1.8~2.0 eV separation for bulk and thin film BFO using near edge x-ray absorption fine structure technique and electron energy loss spectroscopy^28^,^29^,^31^. While there is no significant difference found within the pre-edge region between Fig. 7(a) and (b) (except for slightly higher A’/A intensity ratio for *m*-BFO, which is commonly found in BFO thin films^28^,^29^,^30^), a significant difference is found for the B’/B intensity ratio in the post-edge region between Fig. 7(a) and (b); indeed, while B’ intensity is higher than B in Fig. 7(a), it is much lower than B in Fig. 7(b). This indicates a substantial difference in the Fe-O bonding states resulting from different coordination geometries for the Fe atoms in *r*-BFO and *m*-BFO. Note that the aforementioned characteristics for *r*-BFO matches those reported previously for bulk^28^ and thin film *r*-BFO^25^ in which Fe atom has octahedron bonding geometry with nearest O neighbors as shown in Fig. 8(a). On the other hand, the octahedron bonding geometry between Fe and O atoms changes to a pyramidal one in *m*-BFO, as shown in Fig. 8(b). This is considered to be the reason for the dramatically different B’/B ratios between Fig. 7(a) and (b). Our results are, thus, consistent with previous EEL spectroscopy studies of monoclinic^22^,^31^, and pseudotetragonal^29^ phases from pulsed laser deposition-grown BFO thin films.

### Summary

In summary, crystal and electronic structures of a ~380 nm BFO film grown on a LAO substrate were studied using advanced TEM techniques and the first-principles calculations. BF TEM images reveal that the BFO film consists of two zones, i.e., zone I (=*m*-BFO) and zone II (=*r*-BFO), with different crystal structures. Multiple zone axes ED analysis combined with structure factor calculation readily reveal that crystal structure in zone II is rhombohedral. In contrast, resolving the structure of zone I requires the aid of first-principles calculations to unveil a new orthorhombic-like monoclinic phase with space group of *Cm* (=8). The atomic resolution HAADF image at the interface between LAO and zone I (=*m*-BFO) show a coherent interface with no sign of significant lattice distortion. Atomistic models constructed based on the epitaxial relationship found by ED and structure factor analysis reveal that the lattice mismatch at the interface between *m*-BFO and LAO substrate is ~0.0%, indicating that *m*-BFO, rather than *r*-BFO grows, on LAO since *m*-BFO can more efficiently minimize lattice mismatch with LAO. While the O *K*-edge EEL spectrum from *r*-BFO is consistent with that from bulk BFO, that from *m*-BFO shows drastic differences in the post-edge region, which is considered to be associated with the peculiar bonding geometry, i.e., pyramidal, between iron and oxygen atoms in *m*-BFO.

## Methods

A BFO thin film was grown on a (100) LAO substrate to a thickness of ~380 nm using ultrahigh vacuum (<2 × 10^−6^ Pa) rf magnetron sputtering at 550 °C. Cross-sectional samples for TEM analysis were prepared by dual beam focused ion beam technique, FEI Nova 600, followed by low energy ion polishing, Fischione 1040 Nanomill, with 0.5 kV of Ar ion to minimize beam damage effect. Two different TEM systems operated at 200 kV acceleration voltage were implemented: (1) a JEOL JEM-2100F equipped with Gatan Orius 833 CCD camera for BF TEM images and ED patterns (located in Analytical and Diagnostics Laboratory at State University of New York at Binghamton) and (2) a FEI Titan^2^ G2 80–200 electron probe aberration-corrected scanning transmission electron microscope equipped with Gatan Enfinium spectrometer used to record HAADF-STEM images and EEL spectra (located in ER-C, Forchungszentrum Jülich, Germany).

For the first-principles calculations, we used density functional theory as implemented in the VASP code^32^,^33^. For the *Cm* phase of BFO, the C-type magnetic ordering for Fe spins is considered in our calculations, following the usual approach that was followed, e.g., in ref. 20. All our simulations were done for a 40-atom supercell that can be viewed as a 2 × 2 × 2 repetition of the elemental 5-atom perovskite unit. The energy cut-off and k-point mesh were selected as 500 eV and (4, 4, 4), respectively. The PBEsol^34^ PAW potentials were used in the calculations, with the electronic configurations of 5*d*^10^6*s*^2^6*p*^3^ for Bi, 3*s*^2^3*p*^6^3*d*^7^4*s*^1^ for Fe, and 2*s*^2^2*p*^4^ for O, respectively. A “Hubbard U” correction was used for a better treatment of iron’s 3*d* electrons^35^, with the effective Hubbard U value of 4.0 eV. The crystal structures are relaxed until the force on each atom is less than 0.005 eV/Å.

## Additional Information

**How to cite this article:** Bae, I.-T. *et al*. Elucidation of crystal and electronic structures within highly strained BiFeO_3_ by transmission electron microscopy and first-principles simulation. *Sci. Rep.* **7**, 46498; doi: 10.1038/srep46498 (2017).

**Publisher's note:** Springer Nature remains neutral with regard to jurisdictional claims in published maps and institutional affiliations.

## Supplementary Material

Supplementary Figures

## Figures and Tables

**Table 1:** The *Cm* BiFeO_3_ crystal structure obtained from first-principles calculations.

SG:*Cm*(8)	*a* = 9.26220 Å *b* = 7.58214 Å *c* = 3.79107 Å				
	α = γ = 90^°^; β =~90^°^				
Atom	Wyc.	*x*	*y*	*z*	occupancy
Bi	2*a*	0.49446	0.00000	0.00348	1.00000
Bi	2*a*	0.00170	0.00000	−0.07107	1.00000
Fe	4*b*	0.28226	0.24726	0.51344	1.00000
O	2*a*	0.36427	0.00000	0.51509	1.00000
O	2*a*	0.82589	0.00000	0.53481	1.00000
O	4*b*	0.08420	0.21828	0.55360	1.00000
O	4*b*	0.34783	0.23674	0.01673	1.00000
