# Supplementary material for: Elucidation of crystal and electronic structures within highly strained BiFeO3 by transmission electron microscopy and first-principles simulation
Source: Sci Rep. 2017 Apr 19;7:46498. doi: 10.1038/srep46498 (PMC5395957; doi:10.1038/srep46498)
Supplement: Supplementary Figures [file srep46498-s1.pdf]

Supplementary Information

Elucidation of crystal and electronic structures within highly strained BiFeO3 by transmission electron microscopy and first-principles simulation

In-Tae Bae[1,*], András Kovács[2], Hong Jian Zhao[3], Jorge Íñiguez[3], Shintaro Yasui[4], Tomohiro Ichinose[5], & Hiroshi Naganuma[5,6]

[1]Small Scales Systems Integration and Packaging Center, State University of New York at Binghamton, Binghamton, New York 13902, USA. [2]Ernst Ruska-Centre for Microscopy and Spectroscopy with Electrons (ER-C), Peter Grünberg Institute, Forschungszentrum Jülich, Jülich 52425, Germany. [3]Materials Research and Technology Department, Luxembourg Institute of Science and Technology (LIST), 41 rue du Brill, L-4422 Belvaux, Luxembourg. [4]Department of Innovative and Engineered Materials, Tokyo Institute of Technology, 4259-J2-43, Nagatsuda-cho, Midori-ku, Yokohama, 226-8502, Japan. [5]Department of Applied Physics, Graduate School of Engineering, Tohoku University, Sendai 980-8579, Japan. [6]Unit´e Mixte de Physique, CNRS, Thales, Univ. Paris-Sud, Universit´e Paris-Saclay, 91767 Palaiseau, France. *Correspondence and requests for materials should be addressed to I.-T.B. (e-mail: itbae@binghamton.edu)

Supplementary Figures

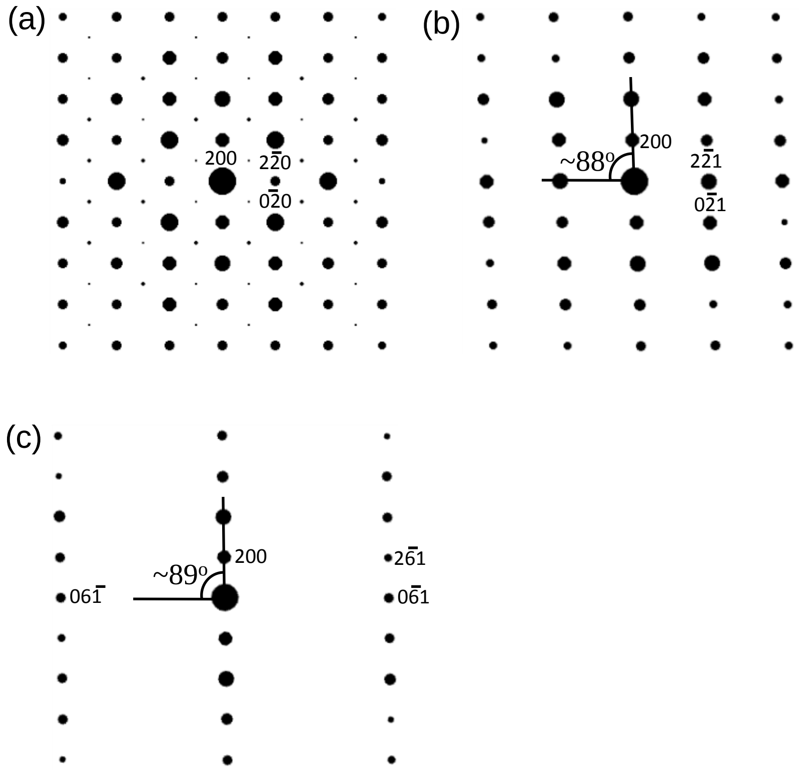

Supplementary Figure S1. Structure factor calculations of monoclinic (space group: *Cm*) BiFeO3 phase anticipated by first-principles simulation in reference 20 along (a) [001], (b) [012], and (c) [016] zone axes.

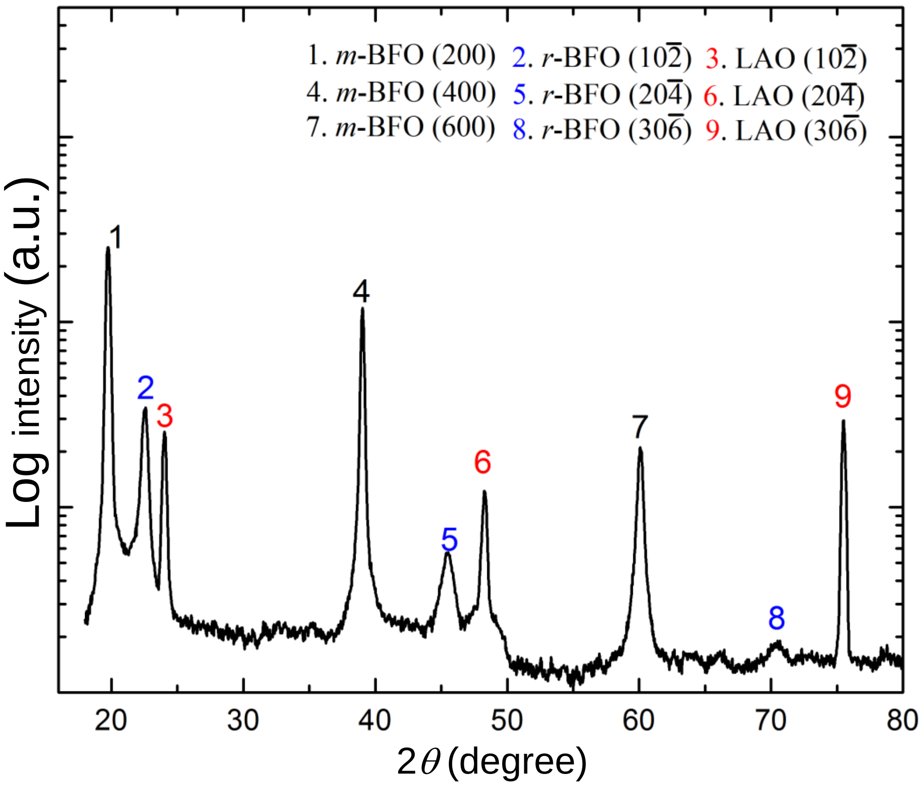

Supplementary Figure S2. XRD θ-2θ scan along surface normal direction for the ~380 nm BFO layer grown on LAO substrate.
